# The Book World of Medicine and Science

**Published:** 1898-12-31

**Authors:** 


### The Book World of Medicine and Science.

Diseases of the Skin: An Outline of the Principles and Practice of Dermatology. By Malcolm Morris, Surgeon to the Skin Department, St. Mary's Hospital. With 10 Coloured Plates and 26 Engravings. New and revised edition. (London: Cassell and Co. 1898. Price 10s. 6d.)

As a small and practical handbook of skin diseases this is likely to prove most useful both to the student and the practitioner. In fact, the rapid sale of the first edition was proof that it met a want, and the new edition is a distinct improvement on the old one. At first sight it might be thought that diseases of the skin, in which the morbid changes must be open to observation and accessible to treatment, ought to be the simplest of all maladies, the most easy of diagnosis, and presenting the most elementary problems in regard to effective treatment. As a fact, the very opposite is the truth, and no class of diseases is more complicated, more puzzling to the practitioner, and more obstinate in its resistance to remedies. The explanation is to be found, as is shown by Mr. Morris, in the fact that the skin is an exposed surface, and that any morbid change which may occur in it is sure to be quickly complicated by the injurious influences of heat and cold, by the friction or pressure of the clothes, by injury of all kinds, and, more especially, by the various sources of irritation furnished by parasitic organisms both animal and vegetable. More especially is the matter complicated by the presence in varying degree in different skins of pus-producing and other micro-organisms, by which the character and results of any inflammation which may take place are liable to be considerably modified. The result is that diseases of the skin, instead of being the simplest, are apt to be the most complex of morbid phenomena, and this probably is the reason why different observers have been led to produce such curiously diverse schemes of classification. The truth is that, as is well put by Mr. Malcolm Morris, "there can be no finality in the classification of cutaneous affections till finality of knowledge of their causation, clinical phenomena, and pathological affinities has been reached," and that is not yet. In the book before us time is not wasted in attempting any new scheme of classification, and all that is attempted in this direction is to arrange the diseases described, as far as that can be done, upon an etiological basis. This is good, but we cannot say that it simplifies matters from a clinical point of view. For example, it involves, including under affections of the skin dependent on nerve disorder, many in the causation of which the nerve is evidently merely the agent of some deeper cause; while, again, some of the most important of the diseases of the skin, such as eczema, psoriasis, pityriasis rubra, and new growths, are left outside the classification altogether, which is unsatisfactory. Practically we fancy that the time has not yet arrived when the student can build up his ideas in regard to skin diseases around any classification. As an after study classification is most important, and is indeed essential to any large views on the subject, but as an aid to the student we may be allowed to doubt whether any such arrangement founded on etiology is likely to be of much service. He has to consider skin diseases as a series of separate maladies, each defined by its own description, and it really is not until he comes to the question of treatment that an etiological classification shows its importance. Mr. Malcolm Morris has, then, done wisely in not allowing himself to give too great prominence to such themes. The strong points in the book before us are the careful clinical descriptions which are given of the various diseases of the skin, and the accurate and practical information in regard to treatment which the author has freely poured out upon its pages from his large experience. It is indeed chiefly in the clearness of the descriptions and the instructive and useful nature of the therapeutic teaching that this book excels. It will, we think, be found a most useful guide both by the student and the practitioner.

Who's Who. 1899. Edited by Douglas Sladen. (London: Adam and Charles Black. Price 3s. 6d. net.)

Among the Christmas annuals which have of late years become essential on the desk and in the library, "Who's Who" has taken a firm place. This it has done by force of merit, and by doing just what other books fail to do. We know there are some who have grumbled at the sort of people who have been selected for notice by the editor of "Who's Who." But herein lies the whole genius of the book. Any-one could give particulars about celebrities whose names are in everybody's mouth, but it requires a knowledge of the world, both extensive and peculiar, to put together such a list as is contained in the book before us, for, in addition to particulars that everyone likes to know concerning celebrities of world-wide fame, it contains a multitude of details, rummaged out from all corners of the earth, about all sorts of people, of whose existence one may, in many instances, have never heard. Yet all these men have made their mark in some career or another, and one never knows at what minute one may want to know all about them and their doings. It is, no doubt, a saddening thought to men who have made a name and are looked upon as leaders in their own departments, to remember that there are 8,000 other people just as good; still, the very vastness of the world of men of note makes it all the more necessary to have some such mutual introduction as is given by "Who's Who." The whole book is very well done, and the new edition is better than any that have gone before, the various tables and other forms of information which precede the biographies having been extended and much improved. To many persons the list, giving the proper pronunciations of peculiarly pronounced proper names, a list which has this year been extended from about 250 to 1,000 entries, would alone be worth the price of the book.

